# Late-onset neuromuscular disorders in the differential diagnosis of sarcopenia

**DOI:** 10.1186/s12883-021-02264-y

**Published:** 2021-06-25

**Authors:** Fabian Hofmeister, Lisa Baber, Uta Ferrari, Stefan Hintze, Stefanie Jarmusch, Sabine Krause, Peter Meinke, Stefan Mehaffey, Carl Neuerburg, Fabiana Tangenelli, Benedikt Schoser, Michael Drey

**Affiliations:** 1 Department of Medicine IV, Geriatrics, University Hospital, LMU Munich, Munich, Germany; 2 Department of Neurology, Friedrich-Baur-Institute, University Hospital, LMU Munich, Munich, Germany; 3 Department of General-, Trauma- and Reconstructive Surgery, University Hospital, LMU Munich, Munich, Germany

**Keywords:** Sarcopenia, Neuromuscular disease, Amyotrophic lateral sclerosis, Myotonic dystrophy type 2, Inclusion body myositis

## Abstract

**Background:**

Sarcopenia is the age-related loss of muscle mass and strength. Undiagnosed late-onset neuromuscular disorders need to be considered in the differential diagnosis of sarcopenia.

**Aim:**

Based on emblematic case reports and current neuromuscular diagnostic guidelines for three common late-onset neuromuscular disorders, a differential diagnostic approach for geriatric patients presenting with a sarcopenic phenotype is given.

**Methods:**

Patients over 65 years of age with sarcopenia, amyotrophic lateral sclerosis, inclusion body myositis and myotonic dystrophy type 2 were recruited. All patients were assessed for sarcopenia based on the revised European consensus definition. Patients with neuromuscular diseases were diagnosed according to the revised El Escorial criteria and the European neuromuscular centre criteria. Phenotypes and diagnostic criteria for all patients were summarized including their specific histopathological findings.

**Results:**

All patients with neuromuscular diseases were positively screened for sarcopenia and classified as severe sarcopenic by means of assessment. The clinical phenotype, the evolution pattern of weakness and muscle atrophy combined with laboratory finding including electromyography could unquestionably distinguish the diseases.

**Discussion:**

Neuromuscular disorders can manifest beyond the age of 65 years and misdiagnosed as sarcopenia. The most common diseases are inclusion body myositis, amyotrophic lateral sclerosis and myotonic dystrophy type 2. A diagnostic work-up for neuromuscular diseases ensures their correct diagnosis by clinical-, electrophysiological, histopathological, and genetic work-up.

**Conclusions:**

In geriatric patients with a focal or asymmetrical muscular weakness and atrophy, sarcopenia assessment should be extended with patient’s history of disease course. Furthermore, concomitant diseases, analysis of serum creatine kinase, electrophysiological examination, and in selected patients muscle biopsy and gene analysis is needed to rule out a late-onset neuromuscular disorder.

## Background

Sarcopenia is defined as a generalized muscular disease leading to a progressive decrease of skeletal muscle mass, strength and physical performance [1]. Falls, impairment and an increased mortality can be a consequence for those affected [2]. The costs for the healthcare systems are enormous and continue to grow, as the worldwide population is ageing [3]. Many researchers have tried in recent years to elucidate the pathogenesis of sarcopenia, but little is published on possible differential diagnoses (DDs). Several neuromuscular disorders (NMDs), e.g. motoneuron-diseases (MNDs) such as amyotrophic lateral sclerosis (ALS), inflammatory myopathies such as inclusion body myositis (IBM) or inherited muscular diseases such as myotonic dystrophy type 2 (DM2) can start with a late-onset and present with sarcopenia-like symptoms [4–6]. While sarcopenia is age-associated (primary sarcopenia) and often accompanied by comorbidities (secondary sarcopenia), the diagnoses of DM2 and IBM require either gene analysis or a histopathological examination of muscle tissue. The diagnosis of ALS is usually made by the combination of clinical findings and the electrophysiology [7–9]. Aim of our study is the comparison of results of the sarcopenia assessments in most relevant late-onset NMDs and sarcopenic patients by emblematic case-reports. Based on our results and current diagnostic guidelines for NMDs, a differential diagnostic approach for geriatric patients given.

## Methods

### Study participants

From the neuromuscular unit of the department for Neurology (Friedrich-Baur-Institute, University Clinic Munich) we recruited three patients with confirmed MND, IBM, and DM2. The sarcopenic patient was selected from a study with geriatric patients suffering a proximal femur fracture screened for sarcopenia during their hospitalisation for fracture treatment [10]. Exclusion criteria were age younger than 65 years, chronic inflammatory diseases or cancer therapy within the last 5 years. All participants gave written informed consent before enrolment. The study protocol was approved by the Ethics Committee, which belongs to the medical faculty of LMU Munich (IRB-No. 328–15).

### Diagnostic criteria of NMDs

**Fig. 1:**
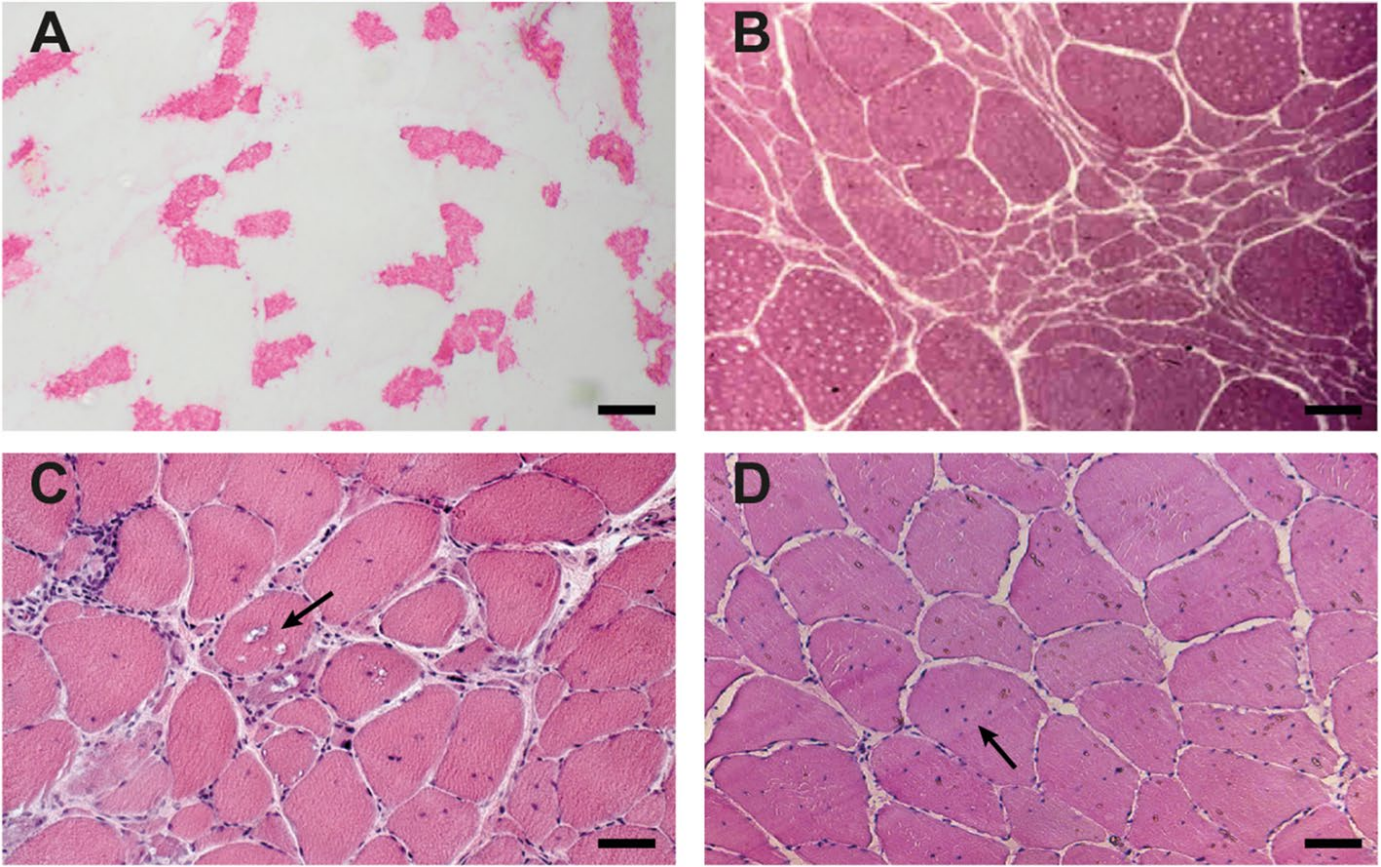
Histopathological findings in muscle biopsies. **A:** Sarcopenia: Vastus lateralis muscle; immunohistochemistry staining for type-2 muscle fibres; Type-2 fibre atrophy; scale bar 100 µm. **B:** ALS: Biceps brachii muscle; haematoxylin–eosin-staining; abnormal shaped atrophic fibres, reticular pattern, neurogenic atrophy; scale bar 50 µm, **C:** IBM: Biceps brachii muscle; haematoxylin–eosin-staining; active inflammation, fibre atrophy, rimmed vacuoles (arrow); scale bar 50 µm. **D:** DM2: Biceps brachii muscle; haematoxylin–eosin-staining; neurogenic atrophy, increased central placed myonuclei (arrow); scale bar 50 µm. Pictures: Olympus CKX53 microscope; Olympus UC90 camera; Olympus cellSens Software

Amyotrophic lateral sclerosismanifests up to the age of 80-years [5, 11]. The disease progressively affects upper- and lower motoneurons leading to muscle spasticity, weakness and muscle atrophy, and early death [11]. Beside the muscular involvement (Table 1), fasciculations, hyperreflexia and elevated serum creatine kinase (CK) levels may be found [7, 12, 13]. The „revised El-Escorial criteria “ (rEEC) (Table 2) are used for the diagnosis, although they only allow a diagnosis to be made in later disease-stages, as several regions have to be affected [7]. Currently, an updated algorithm for making the diagnosis is debated [14]. Additionally, other diseases have to be excluded by electrophysiological examinations and neuroimaging [7]. Diagnostic muscle biopsy can contribute to the final diagnostic work-up, as the muscle specimen may show neurogenic changes with abnormal shaped angulated atrophic fibres in a reticular pattern (Fig. 1B) [15]. Currently elevated neurofilaments markers in the cerebrospinal fluid has been proven useful in assuring the diagnosis at early ALS stages [14].

**Table 1:** Disease characteristics

Disease	Sarcopenia	ALS	IBM	DM2
**Prevalence**^a^	≈ 8500^b^	≈ 5	≈ 5^c^	≈ 10
**Age at onset [years]**	≈ 65	≈ 50–70	≈ 65	≈ 50-70^d^
**Disease-Onset**	symmetrical, generalized, slow progressive	asymmetrical, distal, fast progressive generalized including ventilatory insufficiency	asymmetrical, start frequently weakness of finger flexors, later dysphagia	symmetrical, proximal, axial > distal, cataract, arrhythmia, heart failure
**Disease pattern**				
**Outcome**	Reduced life expectancy by falls and fractures	Lethal within 3–10 years	Disabling within 10–20 years	Normal life expectancy

References: [1, 2, 4, 6, 11, 12, 15–18]

*ALS *  amyotrophic lateral sclerosis, *DM2*  myotonic dystrophy type 2, *IBM*  inclusion body myositis

^a^Prevalence per 100,000 population; ^b^higher prevalence in older people; ^c^in population older than 50 years; d: muscle weakness onset;

**Table 2:** Comparison of the diagnostic guidelines

Disease	Stage	Criteria
**Sarcopenia** (EWGSOP2)	probable sarcopenia	Low muscle strength only
	sarcopenia	Low muscle strength AND Low skeletal muscle mass
	severe sarcopenia	Low muscle strength AND Low skeletal muscle mass AND Low physical performance
**ALS** (rEEC)	suspected ALS	Affection of UMN + LMN in 1 region OR Affection of UMN in ≥ 2 regions
	probable ALS (laboratory supported)	Signs of denervation in the EMG in ≥ 2 limbs AND Affection of UMN + LMN in 1 region OR Affection of UMN in ≥ 2 regions
	possible ALS	Affection of UMN + LMN in 2 regions
	definite ALS	Affection of UMN + LMN in 2 regions + bulbar OR Affection of UMN + LMN in 3 regions
**IBM** (ENMC)	possible IBM	Weakness of finger flexion > shoulder abduction OR Weakness of knee extension ≥ hip flexion AND Age > 45a + disease duration > 1a + elevated CK ≤ 15 × ULN AND At least one, not all pathological features* detectable
	clinically defined-IBM	Weakness of finger flexion > shoulder abduction AND Weakness of knee extension ≥ hip flexion AND Age > 45a + disease duration > 1a + elevated CK ≤ 15 × ULN AND at least one, not all pathological features* detectable
	clinico-pathologically defined IBM	Weakness of finger flexion > shoulder abduction OR Weakness of knee extension ≥ hip flexion AND Age > 45a + disease duration > 1a + elevated CK ≤ 15 × ULN AND All pathological features* 1–3 detectable
**DM2** (ENMC)	ensured DM2	Weakness of iliopsoas muscles, head flexors, myalgia, cataracts, muscle stiffness Molecular genetic testing of DM2 tetranukleotid-repeat expansion

References: [1, 7–9, 13]

a = years; ALS = amyotrohic lateral sclerosis; DM2 = myotonic dystrophy type 2; rEEC = revised El Escorial criteria; ENMC = European Neuromuscular Centre; EMG = Electromyography; EWGSOP2 = revised European Working Group on Sarcopenia and Older People; LMN = lower motoneuron; CK = serum creatine kinase; IBM = inclusion body myositis; ULN = upper limit of normal; UMN = upper motoneuron

^*^pathological features in muscle biopsy: 1. Endomysial inflammatory infiltrates 2. Rimmed vacuoles 3. Protein accumulation or 15-18 nm Filaments 4. Upregulation of major histocompatibility complex class 1

Inclusion body myositis (IBM) is the most common inflammatory myopathy among adults after the age of 50 years [4, 16]. Beside the typical muscular involvement (Table 1), mildly elevated CK levels is found regularly [8]. The guideline of the „European Neuromuscular Centre “ (ENMC) of 2011 is widely used for diagnosis (Table 2) [8]. A histopathological examination of muscle tissue specimen confirms the diagnosis [8]. Typical bioptic findings are a mild to moderate inflammation in combination with myodegenerative findings like rimmed vacuoles and muscle fibre atrophy (Fig. 1C) [8].

While myotonic dystrophy type 1 is the most common adulthood-onset neuromuscular disease before age 45, studies suggest that the prevalence of the later-onset myotonic dystrophy type 2 (DM2) may be at least as high worldwide [19]. Beside the classic proximal and axial muscular involvement (Table 1), the most frequent symptoms of late-onset DM2 are myalgia, myotonia, cataracts, and CK elevation [19]. Muscle biopsy can contribute to the correct diagnosis, as the tissue may show an increase of internalized myonuclei with a lined-up appearance and a type-2 fiber atrophy (Fig. 1D) [9, 15]. Based on the ENMC-guideline of 2010, the combination of phenotype and additional examinations, e.g. EMG myotonia, often directs to a primary DM2 repeat gene analysis, which finally confirms the DM2 diagnosis (Table 2) [6].

### Sarcopenia assessment

For the diagnosis of sarcopenia we used the revised criteria of the “European Working Group on Sarcopenia in Older People” (EWGSOP2) [1]. Muscle mass was assessed by using bioelectrical impedance analysis. The measurement was performed under standard conditions with the patient in a supine position and surface electrodes placed on the wrist and ankle. Appendicular lean mass was estimated using the equation by Sergi et al. [20]. The skeletal muscle mass Index [SMI, (kg/m^2^)] was calculated by dividing appendicular lean mass by squared body height. Threshold for skeletal muscle mass was defined as SMI lower than 5.5 kg/m^2^ in women and 7 kg/m^2^ in men [1]. Handgrip strength (HGS) was assessed with a Saehan DHD-1 Digital Hand Dynamometer, with the patient sitting upright and the forearm held in a 90-degree flexion. Three measurements of both hands were taken and the maximum value was obtained. Thresholds for HGS were 16 kg for women and 27 kg for men [1]. Physical performance was assessed by using the 4 m walking gait speed (GS) test. The higher GS of two measurements was taken. Threshold was 0.8 m/s [1]. Patients with reduced HGS and low SMI were classified as sarcopenic. Sarcopenic patients with reduced GS were classified as severe sarcopenic. Additionally, the sarcopenic participant had an open biopsy of the vastus lateralis muscle during the surgery, as type-2 muscle fibre atrophy seems to play a role in the aging muscle and the development of sarcopenia [10]. Used staining techniques were haematoxylin–eosin and immunostaining (Fig. 1A) [10].

### Common questionnaires

For sarcopenia screening in all patients the SARC-F questionnaire recommended by the EWGSOP2 for case finding was obtained [1]. The questionnaire contains five questions, which quantify the impairment of strength and mobility in daily life activities [21]. Beside the SARC-F questionnaire, the Instrumental Activities of Daily Life (IADL) andnutritional status by the Mini Nutritional Assessment in short form (MNA-SF) were applied [22, 23].

## Results

### Sarcopenic patient

This 85-year-old man suffered a traumatic hip fracture due to a fall in public. He admitted to our trauma department for hip replacement surgery. At the age of 65, he recognized a continuous overall loss of muscle strength. As symptoms worsened, his gait was increasingly unstable. He stumbled more often and had multiple falls within the past years. His participation on daily life activities was increasingly restricted and at the age of 85 years, he regularly relied on the help of his wife for daily activities. As most impacting symptom, he complained about a loss of strength in hands and legs. Peripheral nerve neuropathy, ataxia, extrapyramidal disorder, and other neurological disorders were clinically and by neurophysiological exam excluded. Three days after his hip surgery, the first sarcopenia screening was performed, 20 years after he recognized the first symptoms. He scored 7 points in the SARC-F questionnaire, 10 points in the MNA®-SF and 4 points in the IADL. HGS and SMI were reduced to 19.4 kg and 6.6 kg/m^2^ (Table 3). Sarcopenia was diagnosed. The CK level was within normal range (96 U/l). The examination of muscle tissue revealed a normal histology with subsarcolemmal placed myonuclei and an increase in fiber size variability. There was no evidence of glycogen or fat storage, no vacuoles, or inflammation. The mitochondrial distribution was normal and there were less than 5% cytochrome-C negative fibers. The fiber size variability reflects a muscle atrophy of type-2 fibres. The mean type-1 and type-2 fibre diameter was 74.0 µm and 39.6 µm, respectively (Fig. 1A) [10].

**Table 3:** Patients characteristics and results from sarcopenia assessment

	**Sarcopenia** *n* = 1	**ALS** *n* = 1	**IBM** *n* = 1	**DM2** *n* = 1	**Thresholds** [1, 20–22, 24]
Age [years]	85	68	80	80	
Female	No	No	Yes	No	
BMI [kg/m^2^]	22.5	16.6	19.5	30.9	< 23 = underweight 23–30 = normal > 30 = overweight
Diagnostic delay^a^ [years]	20	7	8	5	
IADL-Score	4	2	5	0	8 = highly independent 0 = highly dependant
MNA®SF-Score	10	6	9	10	> 11: normal 11–8: risk of malnutrition ≤ 7: malnutrition
SARC-F Score	7	10	9	6	≥ 4 possible sarcopenia
Maximum HGS [kg]	19.4	21.2	7.8	20.4	Female: 16; Male: 27
HGSo [kg]	19.2	15.4	4.7	20.0	
HGSo / maximum HGS [%]	99	73	60	98	
SMI [kg/m^2^]	6.6	5.5	3.9	6.9	Female: 5.5; Male: 7.0
Gaitspeed [m/s]	^b^	0.4	0.1	0.3	0.8
CK [U/L]	96	410	390	160	Reference < = 169

*BMI* body mass index, *DM2* myotonic dystrophy type 2, *HGS* hand grip strength, *HGSo* and grip strength, other Hand, *IADL* instrumental activities of daily living, *MNA®-SF* mini nutritional assessment short form, *ALS* amyotrophic lateral sclerosis; SMI = skeletal muscle mass index, *CK* serum creatine kinase, *IBM* inclusion body myositis

^a^time from first reported symptoms to diagnose; ^b^ due to surgery no valid gait speed assessment possible

### ALS patient

At the age of 60-years symptoms started with minor weakness of the left foot flexors and extensors. In the following years his symptoms were progressive, the disease affected muscles of the distal upper extremities, the extensor muscles of his back. Later respiratory muscles became affected, leading to global respiratory insufficiency and the need of a ventilatory support. Seven years later, he finally was referred to the neuromuscular centre. The electromyography of extremities showed a neurogenic pattern with pathological spontaneous activity in all extremities. In the diagnostic work-up, the clinical and electrophysiological evaluation guided the diagnosis of ALS. Our patient manifested as a predominant lower motoneuron disease affection (progressive muscle atrophy, PMA-ALS). This ALS phenotype represents about 15% of all ALS patients and has a less progressive course of the disease [25]. There was no indication for performing an additional diagnostic muscle biopsy. One year later in the clinical examination showed a generalized muscular atrophy and weakness combined with a reduced muscle tone. Loss of function was dominant in both foot flexors (medical research council scale (MRC) grade 0/5) and axial lumbar muscles. His upper extremities showed a milder decrease of muscle strength and function (MRC grade 4–5/5). Reflexes were brisk. In the physical performance test the patient was unable to stand on his toes or heels. The CK was elevated to 410U/l (Table 3). No bulbar involvement was found. The sarcopenia screening was performed eight years after symptom onset and one year after confirming the ALS diagnosis. He scored 10 points in the SARC-F questionnaire, 6 points in the MNA®-SF and 2 points in the IADL. HGS, SMI and GS were reduced to 21.2 kg, 5.5 kg/m^2^ and 0,4 m/s (Table 3). Severe sarcopenia was diagnosed.

### IBM Patient

In 2008, this today 80-year-old women suffered from a traumatic hip fracture. Hip replacement was necessary. Within the next 8 years, she recognized a slowly progressive decrease of her lower extremity muscle strength and physical performance, but blamed this to prior hospitalisation and surgery. At age 76, she consulted the neuromuscular centre for a diagnostic work-up. A muscle biopsy was taken and revealed an active inflammation, fibre atrophy, and rimmed vacuoles (Fig. 1C). Together with the clinical evaluation, the diagnosis of an IBM was made. Two years later, the disease had progressed by dysphagia and to the upper limbs, here predominantly weakened her finger flexors. At the clinical examination at the age of 78 years she presented with a muscle atrophy of her distal extremities. Her knee flexors and extensors, and her foot flexors and extensors showed the severest decline in physical performance (MRC grade 1–3/5). The strength in proximal and distal muscles of her upper extremities was also highly reduced (MRC grade 3/5). She hardly could use her hands by the excessive weakness of her finger and hand flexors. Muscle reflexes were absent. In physical performance tests she showed major difficulties in standing up from a sitting position. The CK was elevated to 390 U/l (Table 3). Sarcopenia screening was performed 10 years after symptom onset and 2 years after confirming the IBM diagnosis. She scored 9 points in the SARC-F questionnaire, 9 points in the MNA®-SF and 5 points in the IADL. HGS, SMI and GS were reduced to 7.8 kg, 3.9 kg/m^2^ and 0,1 m/s (Table 3). Severe sarcopenia was diagnosed.

### DM2 Patient

This 80-year-old man complained about pain in his legs during the night for 10 years. Three years later, he recognized progressive weakness of his proximal lower extremities, especially while climbing a staircase. In the further course of the disease, other muscle groups of the upper extremities, e.g. the neck flexor muscles and the ventilation became affected. The patient introduced himself to the neuromuscular centre five years after his first symptoms. In the diagnostic work-up a muscle biopsy was taken and revealed signs of a neurogenic atrophy with increased central placed myonuclei as the pathological hallmark of DM2 (Fig. 1D). The DM2 gene analysis confirmed the DM2 diagnosis. At the age of 85 years, the clinical examination showed an atrophy of the shoulder girdle muscles. Muscle weakness and atrophy was prominent in neck flexor muscles (MRC grade 2/5), proximal muscles of upper extremities (MRC grade 3–4/5) as well as hip flexors (MRC grade 2/5) and foot-extensors (MRC grade 3–4/5). Truncal axial muscles were also affected, however the hand muscles were spared. The reflexes were normal in upper extremities and reduced in lower extremities. In the physical performance test, he had major difficulties when standing up from lying or sitting position. The CK level was between 160 and up to 620 U/l (Table 3). The sarcopenia screening was performed 8 years after symptom onset and 3 years after confirmation of the diagnosis. He scored 6 points in the SARC-F questionnaire, 10 points in the MNA®-SF and 0 points in the IADL. HGS, SMI and GS were reduced to 20.4 kg, 6.9 kg/m^2^ and 0,3 m/s (Table 3). Severe sarcopenia was diagnosed.

## Discussion

All patients with NMDs would have been wrongly classified as severe sarcopenic and positively screened for sarcopenia by the SARC-F questionnaire. Thus, geriatric patients suffering common late-onset NMDs may be misdiagnosed as sarcopenic and their NMD remains undiagnosed. A reliable diagnosis can only be made when clinicians are aware of important differential diagnoses and their diagnostic guidelines.

The presented NMDs are generally rare diseases compared to sarcopenia. Nevertheless, studies suggest, that the prevalence of DM2 and IBM may be higher than expected [19, 26]. This could be partially related to the fact, that older patients suffering from NMD with sarcopenia-like symptoms are examined and treated primarily by General Practitioners or Geriatricians. They might neglect and miss awareness of potentially late-onset neuromuscular disorders. Our report is based on emblematic cases, therefore we cannot report on any prevalence data of NMDs among geriatric or sarcopenic patient cohorts. Beyond neuromuscular involvement, extramuscular manifestations like heart and lung disease, cataracts and other eye diseases, or isolated dysphagia need to be considered in the spectrum of neuromuscular disorders at advanced ages, especially in DM2. Based on one of the few reports on myopathies in aged patients, Echaniz-Laguna reported of 15 patients older than 70 years. Of these 15 elderly patients, 50% had inflammatory myopathy, 32% had genetically determined myopathy, 16% had myopathy of unspecified cause, and 2% had toxic myopathy. Elderly patients more frequently presented with myalgia, inflammatory myopathy, and cancer [24]. Therefore, our report tries to increase awareness of sarcopenia as important neuromuscular differential diagnosis.

Our participants with IBM and DM2 were both over 75-years old, when the diagnosis of their NMD was confirmed (Table 3). The diagnostic delay encompasses months for ALS, years for IBM or more than a decade for DM2 [4, 11, 19]. A high age at disease onset in combination with a diagnostic delay makes it possible, that patients with undiagnosed NMDs are among geriatric patients.

Treating physicians should consider DDs when a focal or asymmetrical muscular involvement is observed. An asymmetrical affection at disease onset is typical for IBM, here especially the weakness of the finger flexors and the vasting of the proximal vastus lateralis muscles, while in ALS the weakness pattern is more generalized and fast progressing [4, 13]. The difference in HGS of both sides was greatest in our participants with IBM and ALS (60% and 73%, Table 3). In DM2, the proximal weakness e.g. for climbing a staircase is a clinical hallmark, especially if combined with myalgia and stiffness. Unexplained side-differences in HGS found during sarcopenia screening might give an indication of a different diagnose than sarcopenia. While the diagnosis of sarcopenia according EWGSOP2 requires values below specific thresholds, weakness or reduced muscular functionality in NMDs is more often determined by assessments based on the MRC-scale [1, 6, 8]. As the current sarcopenia screening algorithm do not include further assessment in case of an isolated or asymmetrical affection of certain muscle groups, additional examination methods should be considered. An examination by MRC-Scales would—combined with an evaluation of cranial nerves and reflexes—not only be able to detect an asymmetrical or isolated involvement of certain muscle groups, but also an affection of upper and lower motoneurons. If the assessment provides indications of an NMD, additional diagnostics – like CK determination, an electromyography, the evaluation of muscle tissue or genetic analysis – could be arranged.

Since elevated CK occurs inconsistent in the investigated NMDs (Table 3), the distinction power is rather limited [4, 7, 19]. However, lower to borderline CK levels are found commonly in older adults, possibly due to sarcopenia [27]. Thus, a differentiation between sarcopenia and NMDs by CK levels might be possible.

The electromyography reveals signs of an active and chronic denervation in ALS, also in clinically not affected muscles [7]. For the differential diagnosis of ALS, electromyography might be helpful besides CK measurement if the clinical syndrome is suggestive for a motoneuron disease.

In DM2, myotonia can be detected more securely by electromyography than by neuromuscular examinations. An electromyography also contributes to the diagnosis of IBM, however electromyography was removed from the current guidelines partially due to commonly found neurogenic patterns, leading to misdiagnoses of MNDs [8]. Muscle biopsy contributes to ensure or exclude certain NMDs, as reflected in our histopathological findings (Fig. 1) [7, 15].

It should be noted, that no curative therapies are available yet for the investigated NMDs [8, 9, 11]. Nevertheless, a retrospective study by Miró et al. [28] showed muscular diseases in up to 35% of people aged 65 plus, who had a muscle biopsy due to weakness or an elevated CK. It remains unclear in how many patients classified as sarcopenic the diagnosis of a treatable muscular disease is missed.

In the additionally performed questionnaires, none of our participants achieved full scores. Interestingly, the DM2 patient showed the lowest IADL and the highest MNA®-Score, indicating a high impact of his disease on independence and a lower personal risk of malnutrition. If dysphagia is present, swallowing studies with the use of fiberoptic endoscopic evaluation of swallowing (FEES) for the differential diagnosis is warrant. He was overweight (all others underweight) according the age-adjusted BMI by Winter et. al [17]. The more prevalent obesity in DM2 contrasts the cachexia that occurs in ALS and IBM within months or years due to their different neurogenic or inflammatory pathogenesis [4, 11, 18]. The correlation of a more severe decline of muscular strength with higher fat mass in DM2 coincides with observations of IADL-disability in obese sarcopenic patients [18, 29]. Thus, the overweight of our DM2 patient might contribute to his severe functional restrictions. Finally, malnutrition seems to play a role in sarcopenia and our investigated NMDs as well, as dysphagia is reported as a possible symptom [1, 6, 13, 26]. Malnutrition, or at least the risk of malnutrition, was assessed in all patients. Whether this fact contributes to the development of a sarcopenic phenotype in NMDs or is expression of a more severe and rapid muscular affection is unclear.

## Conclusion

Our data show that geriatric patients with specific NMDs may present with symptoms similar to sarcopenia. In patients with unexplained focal or asymmetrical reduced muscle strength and atrophy, assessment of MRC-scales and the examination of cranial nerves and muscular reflexes should be performed. If results of these examinations strengthen the suspicion of a NMD, CK measurement should follow. In patients with repeatedly elevated CK levels, which cannot be explained otherwise, an electromyography should be considered next. This may provide evidence of a NMD by showing myotonia, fasciculations or a neurogenic-/myopathic pattern. If the electromyography reveals no clear indications for a certain NMD, a diagnostic muscle biopsy should be taken into consideration. This may ensure the diagnosis of IBM, other muscular diseases or give hints for DM2. Finally, genetic work-up, including testing for the DM2 repeat expansion should be considered and a referral to a special neuromuscular centre is advised.

## Data Availability

All data generated or analysed during this study are included in this published article.

## Abbreviations

DD: Differential diagnosis
DM2: Myotonic dystrophy type 2
rEEC: Revised El Escorial Criteria
ENMC: European Neuromuscular Centre
EWGSOP2: European Working Group on Sarcopenia in Older People revised
GS: Gait speed
HGS: Handgrip strength
IADL: Instrumental Activities of Daily Living
MNA®-SF: Mini Nutritional Assessment short form
MND: Motoneuron disease
MRC: Medical research council
NMD: Neuromuscular disorder
ALS: Amyotrophic lateral sclerosis
CK: Serum creatine kinase
IBM: Inclusion body myositis
SMI: Skeletal muscle mass index
